# Insights Derived From Text-Based Digital Media, in Relation to Mental Health and Suicide Prevention, Using Data Analysis and Machine Learning: Systematic Review

**DOI:** 10.2196/55747

**Published:** 2024-06-27

**Authors:** Colm Sweeney, Edel Ennis, Maurice D Mulvenna, Raymond Bond, Siobhan O'Neill

**Affiliations:** 1 Department of Psychlogy Ulster University Coleraine United Kingdom; 2 School of Computing Ulster University Belfast United Kingdom

**Keywords:** mental health, machine learning, text analysis, digital intervention

## Abstract

**Background:**

Text-based digital media platforms have revolutionized communication and information sharing, providing valuable access to knowledge and understanding in the fields of mental health and suicide prevention.

**Objective:**

This systematic review aimed to determine how machine learning and data analysis can be applied to text-based digital media data to understand mental health and aid suicide prevention.

**Methods:**

A systematic review of research papers from the following major electronic databases was conducted: Web of Science, MEDLINE, Embase (via MEDLINE), and PsycINFO (via MEDLINE). The database search was supplemented by a hand search using Google Scholar.

**Results:**

Overall, 19 studies were included, with five major themes as to how data analysis and machine learning techniques could be applied: (1) as predictors of personal mental health, (2) to understand how personal mental health and suicidal behavior are communicated, (3) to detect mental disorders and suicidal risk, (4) to identify help seeking for mental health difficulties, and (5) to determine the efficacy of interventions to support mental well-being.

**Conclusions:**

Our findings show that data analysis and machine learning can be used to gain valuable insights, such as the following: web-based conversations relating to depression vary among different ethnic groups, teenagers engage in a web-based conversation about suicide more often than adults, and people seeking support in web-based mental health communities feel better after receiving online support. Digital tools and mental health apps are being used successfully to manage mental health, particularly through the COVID-19 epidemic, during which analysis has revealed that there was increased anxiety and depression, and web-based communities played a part in reducing isolation during the pandemic. Predictive analytics were also shown to have potential, and virtual reality shows promising results in the delivery of preventive or curative care. Future research efforts could center on optimizing algorithms to enhance the potential of text-based digital media analysis in mental health and suicide prevention. In addressing depression, a crucial step involves identifying the factors that contribute to happiness and using machine learning to forecast these sources of *happiness*. This could extend to understanding how various activities result in improved happiness across different socioeconomic groups. Using insights gathered from such data analysis and machine learning, there is an opportunity to craft digital interventions, such as chatbots, designed to provide support and address mental health challenges and suicide prevention.

## Introduction

### Background

Text-based digital media platforms have revolutionized communication and information sharing, offering valuable opportunities to gain insights into various domains, including mental health and suicide prevention.

Social media platforms have become significant sources of data for studying mental health and suicide prevention, where researchers have explored the potential of using platforms such as X (X Corp), formerly known as Twitter (Twitter, Inc) and Facebook (Meta Platforms, Inc) to gain insights into individuals’ mental well-being, detect mental health concerns, and identify suicide risk factors. For example, Coppersmith et al [1] developed a machine learning model to detect signals related to depression in user posts on Twitter, achieving promising results. In addition, De Choudhury et al [2] analyzed Facebook posts to identify individuals at risk of depression, demonstrating the feasibility of using social media data for mental health monitoring. Research methods involve various techniques, including sentiment analysis, topic modeling, and natural language processing (NLP), to analyze large volumes of data and identify patterns and trends. For instance, Park et al [3] applied sentiment analysis to examine suicide-related tweets and identified specific linguistic features associated with suicidal ideation. Sik et al [4] used topic modeling to identify mental health–related topics in web-based forums, facilitating targeted interventions and support. In addition, Burnap et al [5] used NLP techniques to analyze web-based content and identify individuals expressing suicidal ideation, which could enable timely interventions.

Data analysis and machine learning techniques have been used for detecting mental health issues and identifying individuals at risk of suicide, where these sophisticated techniques could enhance clinical decision-making in relation to suicide [6]. Some researchers have explored the use of predictive models to assess suicide risk factors and facilitate early intervention. For example, O’Dea et al [7] developed a predictive model using machine learning algorithms to identify suicide attempt risk among social media users, highlighting the potential for targeted prevention strategies. Data analysis can also be used to provide a valued understanding of factors associated with suicide and mental health, which are not easily identifiable. These insights can then be used to develop strategies for prevention and intervention. For example, data analysis can identify potential underlying causes and risk factors associated with suicide, which can then lead to the development of interventions for susceptible groups. Finally, data analysis can also be used to analyze the effectiveness of current prevention efforts to improve targeted interventions and strategies.

### Objectives

With the rise in the use of smartphones, digital interventions have been able to offer a solution to address the increasing demand for mental health services [8] and to relieve certain barriers in mental health provision, such as the stigma around accessing psychological health services and geographic isolation [9]. This paper presents a systematic review of the research on the application of machine learning and data analysis to text-based digital media data in relation to mental health and suicide prevention to help answer the following research question: How can machine learning and data analysis be applied to text-based digital media data to understand mental health and aid suicide prevention?

## Methods

### Search Strategy: Electronic Database Search

A systematic literature search was performed for articles published from January 1, 2013, to July 10, 2023, and was conducted using 4 databases, namely Web of Science, MEDLINE, Embase (via MEDLINE), and PsycINFO (via MEDLINE), using the following search terms, which were adapted for each database: (mental health OR depression OR suicide) AND (machine learning OR deep learning OR artificial intelligence) AND (text analysis OR text mining OR data analysis) AND (digital intervention OR digital mental health). Retrospective searches were conducted (using the same criteria) using both PubMed and Scopus databases to extend the research to bigger databases. However, no new relevant papers were detected. The complete search strings are included in Multimedia Appendix 1. CS performed the literature search. EE, MDM, and RB discussed and verified the inclusion or exclusion criteria. The *Study Selection* section identifies how articles were included in or excluded from this review. These database searches were supplemented by hand-search techniques. An additional manual search was run using advanced search within Google Scholar (date: July 10, 2023). The first 5 pages of search results (n=50 records) were screened based on title, as per PRISMA (Preferred Reporting Items for Systematic Reviews and Meta-Analyses) guidelines [10].

### Study Selection

**Figure 1:**
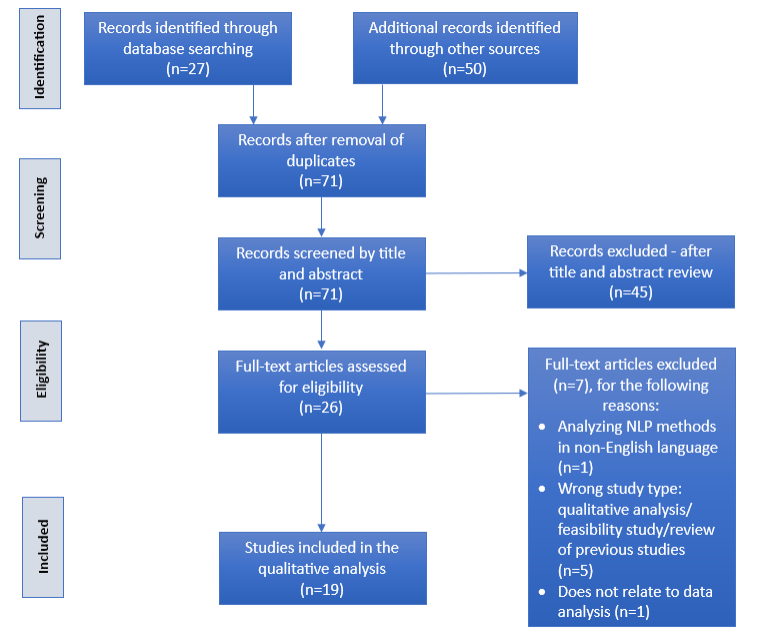
PRISMA (Preferred Reporting Items for Systematic Reviews and Meta-Analyses) flow diagram. NLP: natural language processing.

A total of 27 records were identified according to the search methods explained in the *Search Strategy* section. An additional 50 records were identified by searching Google Scholar articles. Of the 71 unique articles, 45 (63%) were excluded after abstract screening. A full-text review was performed for the remaining 26 (37%) articles according to study inclusion criteria, after which 19 (73%) of these articles were included (Figure 1; Multimedia Appendix 2 [10]). A total of 7 reports failed to meet the stated inclusion criteria. These included papers (1/7, 14%) analyzing NLP methods in a non-English language; papers (5/7, 71%) with a wrong study type, such as qualitative analysis of the use of social media in mental health and teaching mental health intervention in schools or feasibility study or review of previous studies; and papers (1/7, 14%) that did not relate to data analysis. Figure 1 shows a flowchart of the study inclusion process.

### Quality Assessment

An assessment for bias risk was performed using the TRIPOD (Transparent Reporting of a Multivariable Prediction Model for Individual Prognosis or Diagnosis) guidelines [11]. Multimedia Appendix 3 provides more details relating to how the TRIPOD checklist was used and the TRIPOD ratio calculated for the articles relating to prediction and classification (refer to Table S1 in Multimedia Appendix 3 for risk bias results).

## Results

### Types of Analyses to Assess Text-Based Digital Data and Outcomes

This review aimed to determine how machine learning and data analysis can be used to assess text-based digital media data in relation to mental health and suicide prevention. Regarding the type of analysis and outcome measures used within the publications reviewed in this study, machine learning and text-based data analysis were used in 4 (21%) of the 19 studies [12-15]. A total of 3 (16%) studies performed some sort of analysis on survey or questionnaire data [16-19], and 3 (16%) papers analyzed the value of text-based digital media [20-22]. The analysis of digital interventions was the main type of analysis used by Onyeaka et al [23], Vermetten et al [24], and Van Gemert-Pijnen et al [25]. The remaining types of investigations included the analysis of forum or discussion data [26] and longitudinal analysis [27]. Where machine learning was used for prediction within the studies, the outcome metrics were also listed in the table. These include the study by Roy et al [28], who investigated how machine learning approaches could be used to predict suicidal ideation from social media data. They trained a random forest model using neural networks to predict suicide ideation status with an area under the curve of 0.88. Gu et al [29] used convolutional neural network for text for classifier training and classification, which produced the following scores: precision=0.84, recall=0.84, and *F*_1_-score=0.84. Oyebode et al [30] used 5 different machine learning methods to evaluate mental health apps based on user reviews. The 5 models produced similar scores, with the stochastic gradient descent showing the best performance of the 5 classifiers (Table 1).

**Table 1:** Review of themes, showing study and year, title, population, data volume, and theme.

Study and year	Title	Population	Data	Volume	Type of analysis	Outcome metrics	Theme
Aitken et al [16], 2021	How Much of the Effect of Disability Acquisition on Mental Health is Mediated Through Employment and Income? A Causal Mediation Analysis Quantifying Interventional Indirect Effects Using Data From Four Waves of an Australian Cohort Study	Australian households	Data from the HILDA^a^ survey	10,450 respondents who provided data on disability in the survey	Analysis of survey data using causal mediation analysis	Total causal effect of disability acquisition on mental health was estimated to be a 4.8-point decline in mental health.	Predictors of personal mental health
Khattar et al [19], 2020	Effects of the Disastrous Pandemic COVID-19 on Learning Styles, Activities and Mental Health of Young Indian Students-a Machine Learning Approach	Young Indian students	Web-based survey and questionnaire results	583 students’ responses	Analysis of survey and questionnaire data using association rule mining	Rule 1 produced the following scores: support=0.286, confidence=0.671, and lift=1.454	Predictors of personal mental health
Valdez et al [27], 2020	Social Media Insights Into US Mental Health During the COVID-19 Pandemic: Longitudinal Analysis of Twitter Data	Users of the Twitter (Twitter, Inc) social media platform	Publicly available Twitter data	86,581 tweets	Longitudinal analysis using the VADER^b^ sentiment analysis tool	Negative trajectory in sentiment scores for the user-timeline data	Predictors of personal mental health
Xiao et al [17], 2020	Mental Health of Chinese Online Networkers Under COVID-19: A Sociological Analysis of Survey Data	Users of the Chinese WeChat (Tencent Holdings Limited) network platform	Results of completed questionnaires	Across 3491 participants, 2015 questionnaires were valid	Analysis of survey data using OLS^c^ regression	With 1-unit (SD) increase in SES^d^, depression decreased by a margin of –0.52 (*P*<.001).	Predictors of personal mental health
Roy et al [28], 2020	A Machine Learning Approach Predicts Future Risk to Suicidal Ideation From Social Media Data	Users of the Twitter social media platform	Publicly available Twitter data	512,526 tweets	Machine learning for prediction: RF^e^ model using NN^f^ outputs	RF and NN: AUC^g^: 0.88 (95% CI 0.86-0.90)	Detection of mental disorders and suicidal risk
Simms et al [12], 2017	Detecting Cognitive Distortions Through Machine Learning Text Analytics	Users of the Tumblr (Automattic) microblogging and social networking site	Personal blogs from Tumblr	493 posts	Machine learning and text-based data analysis using logistic regression	Accuracy of the logistic model was 73%.	Detection of mental disorders and suicidal risk
Golz et al [26], 2022	Mental Health-Related Communication in a Virtual Community: Text Mining Analysis of a Digital Exchange Platform During the COVID-19 Pandemic	Almost 700 users of inCLOUsiv, a web-based community platform for mental health	Data from the forums and live discussions were stored in the MySQL database	Data set consisted of 31,764 words	Sentiment analysis of forum and discussion data	72% of the identified sentiments were positive.	Understanding how personal mental health and suicidal behavior are communicated
Castilla-Puentes et al [13], 2021	Digital Conversations About Depression Among Hispanics and non-Hispanics in the US: a Big‐Data, Machine Learning Analysis Identifies Specific Characteristics of Depression Narratives in Hispanics	Hispanic and non-Hispanic population in the United States	Open-source sites, message boards, social networks, and blogs	441,000 unique open-source conversations about depression	Machine learning and text-based data analysis: content analysis	Content analysis shows that 66% of conversations among Hispanic population portray a negative tone as compared to 39% among non-Hispanic population.	Understanding how personal mental health and suicidal behavior are communicated
Liu, and Kong [20], 2021	Why Do Users of Online Mental Health Communities Get Likes and Reposts: a Combination of Text Mining and Empirical Analysis	Users on a super topic community relating to depression	Text was obtained from the super topic community relating to depression	Text data and user data for 49,047 posts in the super topic community relating to depression	Analyzing the value of text-based digital media using the LDA^h^ topic model	Social experience in posts (coefficient=0.368), emotional expression (coefficient=0.353), and the sentiment contained in the text (coefficient=0.002) all had significant positive relationships with the number of likes and reposts	Understanding how personal mental health and suicidal behavior are communicated
Falcone et al [14], 2020	Digital Conversations About Suicide Among Teenagers and Adults With Epilepsy: a Big‐Data, Machine Learning Analysis	Teenagers (aged 13 to 19 years) and adults (aged ≥20 years)	Open-source digital conversations across topical sites, blogs, social network, and message boards	222,000 unique conversations about epilepsy, including 9000 (4%) conversations related to suicide	Machine learning and text-based data analysis: thematic analysis	Higher percentage of adults show a defeatist (given up) attitude compared to teenagers (42% vs 4%).	Understanding how personal mental health and suicidal behavior are communicated
Feuston and Piper [21], 2018	Beyond the Coded Gaze: Analysing Expression of Mental Health and Illness on Instagram	Users of the Instagram (Meta Platforms, Inc) social media platform	Posts relating to mental health from Instagram	>3000 posts	Analyzing the value of text-based digital media using constructivist grounded theory approach	Semistructured interviews with 14 adults	Understanding how personal mental health and suicidal behavior are communicated
Waddell et al [18], 2023	Families’ Experiences of Supporting Australian Veterans to Seek Help for a Mental Health Problem: a Linked Data Analysis of National Surveys with Families and Veterans	Veterans and family members	FWS^i^ with linked MHWTS^j^ data	1217 FWS respondents linked with 1123 MHWTS respondents	Analysis of survey data	53% of the respondents thought seeking help would negatively affect their career, and 63% were afraid to ask for help.	Help seeking for mental health difficulties
Gu et al [29], 2023	An Analysis of Cognitive Change in Online Mental Health Communities: a Textual Data Analysis Based on Post Replies of Support Seekers	Users of web-based mental health communities	Reply data from web-based mental health communities	31,935 replies to comments	Machine learning for prediction using TextCNN^k^	TextCNN Precision: 0.84 Recall: 0.84 *F*1-score: 0.84	Efficacy of interventions to support mental well-being
Onyeaka et al [23], 2021	Use of Smartphones, Mobile Apps and Wearables for Health Promotion by People With Anxiety or Depression: an Analysis of a Nationally Representative Survey Data	Respondents to HINTS^l^ 5	Data from HINTS 5	5438 respondents	Analysis of digital interventions using chi-square tests	Respondents with anxiety or depression were generally more likely than people without anxiety or depression, to report that their smart device had helped them in their discussions with their health care providers (42.7% vs 35.3%; *P*=.03).	Efficacy of interventions to support mental well-being
Chikersal et al [22], 2020	Understanding Client Support Strategies to Improve Clinical Outcomes in an Online Mental Health Intervention	Supporters (of clients) on the Silver Cloud (iCBT^m^) platform	Supporter messages to clients	234,735 supporter messages	Analyzing the value of text-based digital media using association rule mining	Lower word count is more salient in more successful messages.	Efficacy of interventions to support mental well-being
Goldberg et al [15], 2020	Machine Learning and Natural Language Processing in Psychotherapy Research: Alliance as Example Use Case	Therapists and clients attending counseling sessions	Recordings from sessions with clients and therapists	Recordings from 1235 sessions	Machine learning and text-based data analysis using MSE^n^ and Spearman rank correlation	The model that used therapist text and extracted features using TF-IDF^o^ performed the best overall, with an MSE of 0.67 and Spearman rank correlation coefficient of 0.15 (*P*<.001).	Efficacy of interventions to support mental well-being
Oyebode et al [30], 2020	Using Machine Learning and Thematic Analysis Methods to Evaluate Mental Health Apps Based on User Reviews	Users of mental health apps	Reviews by users of mental health apps	88,125 user reviews	Machine learning for prediction using SVM^p^, LR^q^, MNB^r^, SGD^s^, and RF	SVM Precision: 0.8940 Recall: 0.8939 *F*_1_-score=0.8939 LR Precision: 0.8938 Recall: 0.8937 *F*_1_-score=0.8937 MNB Precision: 0.8908 Recall: 0.8908 *F*_1_-score=0.8907 SGD Precision: 0.8945 Recall: 0.8943 *F*_1_-score=0.8942 RF Precision: 0.8769 Recall: 0.8770 *F*_1_-score=0.8769	Efficacy of interventions to support mental well-being
Vermetten et al [24], 2020	Using VR-Based Interventions, Wearable Technology, and Text Mining to Improve Military and Veteran Mental Health	Military members and veterans	Self-narratives collected online	300 self-narratives collected online	Analysis of digital interventions	The variable of the word “family” was found to be the most significant predictor in LIWC^t^.	Efficacy of interventions to support mental well-being
Van Gemert-Pijnen et al [25], 2014	Understanding the Usage of Content in a Mental Health Intervention for Depression: an Analysis of Log Data	Users of the web-based intervention “Living to the Full”	Log data from the web-based intervention system	206 participants	Analysis of digital interventions using linear regression	Linear regression yielded a significant model with log-in quartile as a significant predictor (explained variance was 2.7%).	Efficacy of interventions to support mental well-being

^a^HILDA: Household, Income, and Labor Dynamics in Australia.

^b^VADER: Valence Aware Dictionary and Sentiment Reasoner.

^c^OLS: ordinary least squares.

^d^SES: socioeconomic status.

^e^RF: random forest.

^f^NN: neural network.

^g^AUC: area under the curve.

^h^LDA: latent Dirichlet allocation.

^i^FWS: Family Well-Being Study.

^j^MHWTS: Mental Health Well-Being Transition Study.

^k^TextCNN: convolutional neural network for text.

^l^HINTS: Health Information National Trends Survey.

^m^iCBT: internet-based cognitive behavioral therapy.

^n^MSE: mean squared error.

^o^TF-IDF: term frequency–inverse document frequency.

^p^SVM: support vector machine.

^q^LR: logistic regression.

^r^MNB: multinomial naive Bayes.

^s^SGD: stochastic gradient descent.

^t^LIWC: Linguistic Inquiry and Word Count.

Another study [12] used logistic regression, with a 73% accuracy of the logistic model in detecting cognitive distortions. Linear regression was another method used in predicting depressive symptoms and yielded a significant model as a significant predictor of depression [25]. Machine learning was also used in a psychotherapy research study, where the model that used therapist text and extracted features using term frequency–inverse document frequency performed the best overall, with a mean squared error of 0.67 and Spearman rank correlation coefficient of 0.15 (*P*<.001) [15]. Association rule mining was used in analyzing survey data [19], where the top rule identified an association between strong disappointment with missing events and missing friends in person (support=0.286, confidence=0.671, and lift=1.454) due to the COVID-19 pandemic.

Sentiment was measured for various studies; it was measured as positive for a web-based community platform for mental health [26], and text had a positive score, which correlated with the number of likes [20] of the posts. Another survey [23] found that respondents with anxiety or depression were generally more likely to report that their smart device had helped them in their discussions with their health care providers, compared to respondents that did not have anxiety or depression (42.7% vs 35.3%; *P*=.03). Furthermore, a negative tone was observed in 66% of conversations among Hispanic populations compared to 39% of conversations among non-Hispanic populations [13], and the total causal effect of disability acquisition on mental health was estimated to be a 4.8-point decline in mental health [16]. Moreover, there was a negative trajectory in sentiment scores from a longitudinal analysis of Twitter data during the COVID-19 pandemic [27]. Another study [14] reported a higher percentage of adults with epilepsy showing a defeatist attitude compared to teenagers with epilepsy (42% vs 4%). In a family well-being study, 53% of respondents thought seeking help would negatively affect their career, and 63% were afraid to ask for help [18]. The results of a questionnaire to establish the mental health of Chinese web-based networkers found that with an increase in socioeconomic status, depression decreased by a margin of –0.52 (*P*<.001) [17].

Having identified the 19 papers for further analysis, we attempted to identify any themes within these papers. This involved an initial in-depth review to become familiarized with the text, and using simple coding to highlight sections of the texts that best describe the content, we were able to identify shorthand labels or codes, for example, prediction and detection of mental disorders and suicide risk. From the coding, we were then able to identify 5 themes as to how machine learning and data analysis techniques could be applied. The themes are outlined with the number of papers per theme in Table 2.

**Table 2:** Themes and number of papers per theme (N=19).

Theme	Name	Papers, n (%)
1	As predictors of personal mental health	4 (21)
2	To detect mental disorders and suicidal risk	2 (11)
3	To understand how personal mental health and suicidal behavior are communicated	5 (26)
4	To identify help seeking for mental health difficulties	1 (5)
5	To determine the efficacy of interventions to support mental well-being	7 (37)

Details of the 19 papers that were reviewed, including the author, year, title, population studied, data volume, and main themes, are provided in Table 1. The themes are further expanded in the subsequent sections.

### Predictors of Personal Mental Health

Personal mental health can be influenced by various factors, such as employment status and income, and various analytical tools have been used to determine sentiment or other predictors of personal mental health. Research by Aitken et al [16] sought to determine the extent to which alterations in employment and income impact mental health. They used logistic regression models specifically for employment and income, considering their conditional relationship with disability acquisition. The analysis technique focused on evaluating the significance of text-based digital media; their findings indicated that 10.6% of the effect of disability acquisition on mental health was explained by changes in individuals’ employment status, but no similar effect was observed through changes in income. This underscores the importance of measures for addressing disability-related mental health disparities, specifically the equalization of employment rates between individuals with and individuals without disabilities to reduce disability-related mental health inequalities.

Research by Xiao et al [17] sought to examine survey data to measure the prevalence of depression symptoms and their correlation with an individual’s socioeconomic status and lifestyle during the COVID-19 pandemic in China. The methodology involved statistical analyses using SPSS (IBM Corp) to evaluate survey data. The findings revealed a noteworthy impact of the pandemic, indicating that respondents experienced more severe mental symptoms when their residential communities were more exposed to SARS-CoV-2. The implications drawn from these findings suggest that mental health conditions among survey respondents varied based on the level of the COVID-19 pandemic severity. Notably, residents in communities with a high severity of the pandemic exhibited more pronounced symptoms of depression and anxiety.

Khattar et al [19] conducted a web-based survey study with the goal of understanding the day-to-day experiences and mental well-being of young students in India during the COVID-19 pandemic. They analyzed survey responses using R (The R Foundation) and Python (Python Software Foundation) to evaluate the mental health of diverse populations during the ongoing COVID-19 pandemic. Their findings revealed that approximately 19.2% of the students expressed weariness with phone use, while 42.9% reported feeling a mix of frustration, profound boredom, anxiety, overwork, and depression. Conversely, 37.9% indicated experiencing emotions such as relaxation, peace, optimism, calmness, hopefulness, and love. This suggests a crucial role for teachers and mentors in providing emotional support to students. They also used association rule mining to analyze the survey data, where the top rule identified an association between strong disappointment with missing events and missing meeting friends in person (support=0.286, confidence=0.671, and lift=1.454) due to the pandemic.

Valdez et al [27] investigated the extent of social media use at the onset of the COVID-19 pandemic to uncover emerging themes from tweets related to COVID-19 and to examine whether sentiments changed in response to the COVID-19 crisis. They used the latent Dirichlet allocation method for topic modeling and the Valence Aware Dictionary and Sentiment Reasoner for sentiment analysis. Their findings indicated that sentiment scores were initially high and stable but exhibited a significant decrease over time, indicating reduced sentiment over the long term.

Various data analysis techniques have been applied as predictors of personal mental health, where the effect of disability acquisition on mental health, for example, was explained by changes to people’s employment but not by changes to income [16]. In relation to the COVID-19 pandemic, the overall emotional state of students during lockdown showed a mix of various moods, with feelings ranging from frustration to boredom to anxiety to depression [17]. In addition, themes emerged from tweets about COVID-19 to highlight the extent to which social media use increased during the onset of the COVID-19 pandemic [19] and how the sentiment changed in response to the pandemic [27]. The pandemic has had a significant impact on mental health, where respondents had more serious mental symptoms when their residential communities exhibited a greater exposure to the spread of SARS-CoV-2 [17].

### Detection of Mental Disorders and Suicidal Risk

Machine learning can be used in the detection of cognitive distortions, which may fuel anxiety, and in the detection of those at risk of suicide. Roy et al [28] developed a model capable of predicting individuals at risk and assessing the likelihood of experiencing suicidal thoughts within a specific time frame. This involved using a random forest model that used output from neural networks to predict binary suicidal ideation status when there is a match with at least one of the word patterns in the ordered word screening, for example, “feeling suicidal.” This study found that the neural network models successfully predicted suicidal ideation even before individuals articulated explicit thoughts of suicide. These findings suggest that there may be potential for predicting suicidal ideation before individuals explicitly express such thoughts, offering opportunities for early intervention and support.

Simms et al [12] demonstrated that machine learning could also be applied to detecting cognitive distortions (eg, the user would be thinking negatively and discounting the positive) from personal blogs. Through the use of the Linguistic Inquiry and Word Count software, this study found that it is feasible to automatically detect cognitive distortions from personal blogs with a relatively high accuracy of 73%. The implications drawn from these findings underscore the potential benefits of continued work in this area for mental health care and psychotherapy. This progress has the potential to lead to lower costs, earlier detection, and more efficient use of counseling time.

These findings show that it is possible to detect cognitive distortions automatically from personal blogs with an accuracy of 73% [12], and this could lead to an earlier detection of anxiety and possible intervention at an earlier stage. Neural network models, which are powerful machine learning tools, have been shown to successfully detect mental disorders and suicidal risk, where certain models were shown to predict suicide ideation even before suicidal thoughts were articulated [28].

### Understanding How Personal Mental Health and Suicidal Behavior Are Communicated

When attempting to understand how personal mental health and suicidal behavior are communicated, machine learning has been used to explore big data from open-source digital conversations with regard to suicidality. The aim of the research by Castilla-Puentes et al [13] was to delve into big data derived from open-source digital conversations among Hispanic populations to determine attitudes toward depression, comparing Hispanic and non-Hispanic populations. The methodology involved the analysis of tone, topic, and attitude relating to depression using machine learning and NLP. This study revealed a notable disparity in attitudes, beliefs, and treatment-seeking behavior between the 2 groups, providing insights into the mindset and attitudes toward depression from a previously unexplored vantage point.

Falcone et al [14] investigated big data derived from open-source digital conversations among teenagers and adults with epilepsy with regard to suicidality. They used NLP and text analytics to reveal that a higher percentage of teenagers, compared to adults, expressed a fear of “the unknown” due to seizures (63% vs 12%), concern about the social consequences of seizures (30% vs 21%), and desire for emotional support (29% vs 19%). In contrast, a significantly higher percentage of adults exhibited a defeatist (“given up”) attitude compared to teenagers (42% vs 4%). The implications of this study suggest that teenagers engage more frequently in web-based conversations about suicide than adults and that there are notable differences in attitudes and concerns between the 2 groups. These distinctions may have implications for the treatment of younger patients with epilepsy.

Liu and Kong [20] sought to identify the factors influencing the number of likes and reposts within a web-based community dedicated to depression. This involved using a combination of text mining and empirical analysis to delve into the factors affecting user engagement, specifically the number of likes and reposts. They found that users within web-based mental health communities exhibit a higher level of attention to topics related to social experiences and emotional expressions. These findings emphasize that understanding the factors influencing the number of likes and reposts in web-based mental health communities can be advantageous for users, facilitating greater support and providing a sense of relief and comfort within the community.

Feuston and Piper [21] integrated manual data collection with digital ethnography (study of human interaction through the internet technologies used) and semistructured interviews to explore how various modes of expression (eg, visual, textual, and oral) contribute to the overall understanding of mental health. By evaluating the value of text-based digital media, they found that individuals adopt a diverse range of practices and use Instagram (Meta Platforms, Inc) features to render their experiences with mental health and illness visible to others. This would have implications for the analysis of user interactions, suggesting an information flow from one person to the next.

Golz et al [26] used the inCLOUsiv platform to identify and interpret the communication patterns and verbal expressions of the users of the platform during the initial lockdown in 2020. The methodology involved analyzing discussions in forums and live chats using text mining, frequency analysis, correlation analysis, n-gram analysis, and sentiment analysis. Their analysis found that the communication behavior of users on the inCLOUsiv platform was characterized by generosity and support, with 72% of the identified sentiments being positive. Users actively engaged with topics such as *corona*, *anxiety*, and *crisis*, sharing coping strategies, which suggest that positive and supportive interactions within mental health–related virtual communities, emphasizing the potential impact of such interactions on the well-being of community members.

When it comes to understanding how personal mental health and suicidal behavior are communicated, it was found that teenagers engage more frequently in web-based conversations about suicide than adults [14] and that the communication behavior of users on a digital exchange platform was supportive and sentiments were mostly positive [20]. Data analysis was also shown to reveal that individuals use a variety of practices and features of social media to make experiences with mental health and illness visible to others [21] and that users of web-based mental health communities were found to be more attentive to the topics of social experience and emotional expressions [20]. Furthermore, help seeking was shown to vary between different populations, where the attitudes, beliefs, and treatment-seeking behavior toward depression showed great disparity between Hispanic and non-Hispanic populations [13]. Finally, in relation to a specific illness, epilepsy, a higher percentage of teenagers were fearful of “the unknown” due to seizures and concerned about the social consequences of seizures, while a significantly higher percentage of adults showed a defeatist (“given up”) attitude compared to teenagers [14].

### Help Seeking for Mental Health Difficulties

An analysis of survey data has been shown to identify help seeking for mental health difficulties. Research by Waddell et al [18] sought to examine survey data to gain insights into the dynamics of help-seeking relationships within veteran families. The findings of the study brought to light that family members of veterans play a significant role in both the initial and ongoing processes of seeking help. However, the study also revealed substantial barriers to help seeking, primarily linked to the military culture. These barriers included the belief that mental health concerns could be self-managed (if recognized), highlighting concerns about potential impacts on careers and the fear of judgment by others. Educating families about identifying early signs of mental health problems is crucial to inform families about the potential mental health risks associated with military careers. This knowledge can then contribute to fostering a supportive environment and breaking down barriers to help seeking within veteran families.

### Efficacy of Interventions to Support Mental Well-Being

The effectiveness of interventions to support mental well-being has also been analyzed using machine learning. Gu et al [29] used NLP technology to identify psychological cognitive changes. Using an emotion dictionary along with Word2vec semantic training, a model was trained to transform labeled text into a vector matrix, and the convolutional neural network for text was used for classifying the labeled text. The findings of the study indicated that posts signaling cognitive change tended to have longer word lengths. In addition, support seekers who had not undergone cognitive change tended to express themselves more in web-based replies. This highlights the potential for supporting individuals with mental health problems, promoting the development of web-based mental health communities, and constructing web-based psychological chatbots.

Research by Goldberg et al [15] used NLP and machine learning techniques to predict one of the most studied process variables in psychotherapy: therapeutic alliance. The methodology involved using Sent2vec to map sentences to vectors of real numbers, and linear regression was then used as the prediction model. The findings of the study revealed that across the 1235 alliance ratings, the mean rating was 5.47 (SD 0.83), indicating a negative slant often found in the assessment of therapeutic alliance. The implications drawn from these findings suggest that machine learning holds promise for predicting observable linguistic behaviors, these models could be trained using human coding as the gold standard, and thorough testing should be conducted using large data sets.

Oyebode et al [30] used sentiment analysis and other machine learning approaches to evaluate 104 mental health apps available on Google Play (Google LLC) and App Store (Apple Inc). By integrating NLP and the term frequency–inverse document frequency weighting technique to vectorize the reviews, supervised machine learning classifiers were used to predict sentiment. The study revealed that the majority of the reviews were positive, indicating that most users found mental health apps to be useful and helpful, emphasizing the importance of ensuring that mental health apps are not only usable and of high quality but also supportive, secure, and noninvasive.

Research by Chikersal et al [22] provided a deeper understanding of how supporter behaviors impact the use of web-based therapy programs. The methodology involved the application of unsupervised machine learning, along with statistical and data mining methods, to analyze complex, large-scale supporter-client interactions. They found that concrete, positive, and supportive feedback from supporters, particularly those referencing social behaviors, were strongly associated with better outcomes. This suggests the importance of identifying effective context-specific support strategies using data for personalized mental health support. This knowledge can contribute to improving the design and implementation of personalized human support in internet-based cognitive behavioral therapy and enhance our understanding of big data in digital health interventions.

Onyeaka et al [23] investigated the use and perceived benefits of digital health tools, identifying the association between the use of digital interventions and the adoption of healthy lifestyle behaviors, and the sociodemographic factors linked to the use of digital tools among individuals with anxiety or depression. Basic descriptive statistics and chi-square tests were used, identifying a notable prevalence of digital interest among individuals with anxiety or depression, with up to 84.7%, 60.6%, and 57.7% of the individuals reporting ownership of smartphones, tablets, and health apps, respectively. These results suggest that digital tools may offer promise for a subset of individuals with mental illness who prefer engaging in technology-based strategies for managing their health.

Vermetten et al [24] investigated the potential use of virtual reality (VR)–based interventions, wearable technology, and text mining to enhance the mental health of military personnel and veterans. Using text mining and the statistical technique of item response theory, they demonstrated that there was a high agreement of 82% with the diagnoses provided by psychiatrists and suggested that the combination of text mining and VR-based interventions holds promise as a valuable tool for psychological and psychiatric assessments in the future.

Van Gemert-Pijnen et al [25] demonstrated how log data could be used to comprehend the adoption of web-based interventions and provide value in improving the incorporation of content in such interventions. By performing a statistical analysis using SPSS, this study showed that pattern recognition could be used to customize the interventions based on use patterns from earlier lessons and act as an aid in supporting the adoption of content essential for therapy. Understanding how participants can derive greater benefits from the intervention and identifying the most effective combination of features can lead to enhancing the effectiveness of web-based interventions.

There are many ways in which data analysis can be used to support mental well-being; for example, textual data analysis can be used to signal cognitive change, where it has been found that the average word length within text is longer for posts that indicate a cognitive or emotional change [29]. Other analysis results indicate a high prevalence of digital interest among people with anxiety or depression [23], and when NLP and machine learning were used to predict therapeutic alliance, the mean rating showed a typical negative skew found in the assessment of the alliance [15]. VR-based interventions, wearable technology, and text mining are expected to be promising tools in psychiatric assessments in the future [24]. Regarding the use of log data to improve the uptake of a web-based intervention, user pattern recognition from earlier lessons can be applied to tailor the intervention and support the uptake of content essential for therapy [25]. For web-based and non–web-based mental health apps, the majority of the reviews from a study of mental health apps available on Google Play and the App Store were positive, showing that most users found mental health apps useful and helpful [30].

## Discussion

### Principal Findings

When attempting to discover useful insights from text-based digital media in relation to mental health and depression, machine learning and data analysis techniques can be applied in many different ways. They can be used as predictors of personal mental health, for example, to measure how an individual’s socioeconomic status can relate to depression. With the increasing prevalence of mental health issues since the COVID-19 pandemic [31] and the need for effective suicide prevention strategies, using data analysis and machine learning techniques in textual digital media data research has demonstrated that the COVID-19 pandemic and its associated restrictions have resulted in increased depression, anxiety, and feelings of loneliness [32], but this sentiment improved following the news of vaccine rollout to defend against the virus [33]. The pandemic has made a big impact on research in this area, where findings show that students’ overall emotional well-being reflected a combination of diverse moods, encompassing feelings of frustration, boredom, anxiety, and being overworked, and experiencing depression during the pandemic. Further themes that emerged from tweets related to the COVID-19 pandemic showed that social media use increased during the onset of the pandemic and that participants of a survey exhibited more pronounced mental health symptoms if their residential communities faced heightened exposure to the spread of SARS-CoV-2.

Machine learning and data analysis techniques can also be used to detect mental ill health and suicidal risk, where neural network models can be used to predict suicide ideation before suicidal thoughts are articulated and to generate models capable of predicting individuals who would be at risk of suicidal thoughts. These tools can also be used to comprehend help seeking for mental health difficulties. Survey data were analyzed to understand help seeking in relation to mental health, identifying that the role of the family is important in encouraging help seeking for war veterans and revealing substantial barriers to help seeking, particularly in relation to the military culture, such as the belief that mental health concerns can be self-managed (if recognized) and a fear of being judged by others.

When attempting to understand how we communicate personal mental health and suicidal behavior, machine learning techniques can be used in diverse ways, such as to explore digital conversations with regard to suicidality and to identify factors influencing the number of likes in a web-based community for depression. Users were shown to exhibit both benevolent and supportive communication behaviors, with predominantly positive sentiments, on a digital exchange platform. When examining a specific illness, epilepsy, it was revealed that a higher percentage of teenagers expressed a fear of the unknown associated with seizures and concern about the social consequences of seizures, and a higher percentage of adults demonstrated a defeatist attitude compared to teenagers. When Instagram was used to better understand how we can communicate personal mental health, it was disclosed that individuals use various practices and features on the platform to make their experiences with mental health and illness visible to others. Finally, seeking assistance was found to differ across different populations, with significant differences in attitudes, beliefs, and the propensity to seek treatment for depression observed between Hispanic and non-Hispanic populations.

Insights from data analysis and machine learning can be used to assist in the development of digital interventions, and the effectiveness of these interventions can be shown to provide support to people living with depression and improve mental well-being. Through textual data analysis, it was determined, for example, that posts signaling cognitive change exhibit longer word lengths and that support seekers who have not undergone cognitive change tend to express themselves more in web-based replies. Similarly, it was found that there was a heightened prevalence of digital interest among individuals with anxiety or depression. NLP and machine learning can also be used to predict therapeutic alliance between the patient and therapist.

When exploring the potential of VR-based interventions integrating wearable technology and text mining to enhance mental health, it emerged that text mining coupled with VR-based interventions is anticipated as a promising tool for psychological and psychiatric assessments in the future. The use of mental health apps was analyzed, which showed that attitudes toward them were mainly positive, indicating that a majority of users find these apps useful and helpful. In the context of understanding the uptake of web-based interventions, pattern recognition was used to tailor individual interventions based on use patterns from earlier lessons, thereby supporting the uptake of content essential for therapy.

### Limitations

This study exhibits limitations in the selection of articles because it used only 4 journal databases (ie, Web of Science, MEDLINE, Embase, and PsycINFO) as well as Google Scholar. Moreover, only articles published in English and related to mental health or suicide, machine learning and data analysis, and digital interventions were included. The search for articles started in March 2023, and the collected articles were published between 2013 and 2023. As some of the researched articles identified some sort of machine learning classification or prediction, we should have considered explainable artificial intelligence to facilitate the understanding of any predictions made by the machine learning models to better understand the models’ behavior. Another limitation involves how the inclusion and exclusion of papers were resolved. Even though CS, EE, MDM, and RB assessed the papers and decided what was to be included or excluded based on the applicability criteria, it was CS who made the final decision about what went into this review.

### Conclusions

In conclusion, this review illustrates that the use of data analysis and machine learning techniques to extract useful insights from text-based digital media related to mental health and suicide prevention holds significant promise. Data analysis and machine learning were used to gain valuable insights; for example, findings show that engagement in web-based conversations relating to depression may vary among different ethnic groups and that teenagers engage in web-based conversations about suicide more often than adults. Another finding was that disability acquisition (which is associated with a deterioration in mental health) was shown to be affected by changes to employment but not income.

The efficacy of digital tools was also analyzed, with machine learning approaches being used to understand users’ opinions regarding mental health apps. Using positive and negative sentiments, it was shown that those with mental illness are digitally connected and are incorporating these tools to manage their health. Predictive analytics was also identified to be able to detect cognitive distortions, which are associated with depression and anxiety, from personal blogs with an accuracy of 73%, while other machine learning models were able to predict the risk of suicidal ideation from social media. The use of modern technology has also been investigated, with the application of VR-based interventions showing promising contributions to the field of military and veteran mental health by developing new approaches to delivering preventive or curative care.

The recent pandemic has also had an influence on this area of research. Analysis was undertaken to try to discover to what extent social media use increased during the onset of the COVID-19 pandemic and to assess how different populations communicated regarding their mental health. It was discovered that virtual communities played an important role in mental health during the pandemic and that social media may be used as a coping mechanism to combat feelings of isolation related to long-term social distancing. Web-based communities also offer great support for people with mental disorders, where the analysis of the number of likes and reposts for posts in web-based mental health communities allowed for these users to gain more support within the community.

Future research could focus on investigating further benefits of textual digital media analysis in mental health and suicide prevention when dealing with depression and, importantly, what makes people happy. Machine learning can be used to predict what are the sources of “happiness” or even how different activities make different socioeconomic groups “happy,” and these insights can then be used to assist in the development of a wide range of digital interventions, such as chatbots.

Ultimately, this systematic review underscores the importance of harnessing advanced analytical methods to derive valuable insights that can lead to improved mental health interventions and enhanced strategies for suicide prevention.

## Appendix

Multimedia Appendix 1 Electronic database search criteria.

Multimedia Appendix 2 PRISMA (Preferred Reporting Items for Systematic Reviews and Meta-Analyses) checklist.

Multimedia Appendix 3 Details relating to the TRIPOD (Transparent Reporting of a Multivariable Prediction Model for Individual Prognosis or Diagnosis) checklist.

## Abbreviations

NLP: natural language processing
PRISMA: Preferred Reporting Items for Systematic Reviews and Meta-Analyses
TRIPOD: Transparent Reporting of a Multivariable Prediction Model for Individual Prognosis or Diagnosis
VR: virtual reality
