# Supplementary material for: Insights Derived From Text-Based Digital Media, in Relation to Mental Health and Suicide Prevention, Using Data Analysis and Machine Learning: Systematic Review
Source: JMIR Ment Health. 2024 Jun 27;11:e55747. doi: 10.2196/55747 (PMC11240075; doi:10.2196/55747)
Supplement: Multimedia Appendix 1 [file mental_v11i1e55747_app1.docx]

**Electronic Database – Search criteria**

mental health or mental health* or mental disorder or mental health symptoms or depress* or anxiety or mental health crisis or self-harm or suicid* or suicide prevention or mental health emergenc* or self-Injurious behavior or self-injurious behaviour or mental ill* or self-harm or self-injury or well being (Title)

and

prediction or machine learning or machine intelligence or data mining or data science or big data or algorithm* or predictive analy* or classifier* or cluster* or deep learning or artificial intelligence or AI or computational intelligence* or pattern recognition or pattern classification or text classification or classification or recommender system* or deep learning or or random forest or decision tree* or naive bayes or bayesian or support vector machine* or SVM or cluster* or neural network* (Title)

and

text analys* or text-base* or data analy* or text mining or natural language process* or text process* or sentiment analys* or information extraction (Title)

and

digital intervention* or mental health intervention* or digital mental intervention* or digital mental health intervention* or digit* mental health* or digital technolog* intervention* or digit* chat or digit* conversation or digital therapeutics* or virtual mental health* or mobile mental health* or online mental health* or computer-based mental health* or internet-based mental health* or e-mental health or help seek* or m-health or mobile health or digital health or e-health (Title)

**Google Scholar – Search criteria**

(mental health OR depression OR suicide) AND (machine learning OR deep learning OR artificial intelligence) AND (text analysis OR text mining OR data analysis) AND (digital intervention OR digital mental health)*

*Note: Google Scholar would not accept full text criteria, so the search criteria was reduced, to the above.
