# Supplementary material for: Insights Derived From Text-Based Digital Media, in Relation to Mental Health and Suicide Prevention, Using Data Analysis and Machine Learning: Systematic Review
Source: JMIR Ment Health. 2024 Jun 27;11:e55747. doi: 10.2196/55747 (PMC11240075; doi:10.2196/55747)
Supplement: Multimedia Appendix 3 [file mental_v11i1e55747_app3.docx]

# Bias assessment

The assessment of bias risk followed the Transparent Reporting of a Multivariable Prediction Model for Individual Prognosis or Diagnosis (TRIPOD) guidelines [1].

Individual TRIPOD ratios were calculated for each article by dividing its TRIPOD score by the maximum possible points based on included features. Bias assessment of the relevant studies included in the systematic review are shown in Supplementary Table 1.

There are 22 primary features and various subitems in this checklist (see Supplementary Table 2), totalling 37 features. A TRIPOD score was created using 1 possible point for each subitem, as proposed by [2]. Each subitem contributed one point to a TRIPOD score, with adherence earning one point and non-adherence zero points. Features not applicable due to study nature were excluded from analysis.

The primary features included title (1 point), abstract (1 point), introduction - background and objectives (2 points), methods - source of data (2 points), methods - participants (3 points), methods - outcome (2 points), methods - predictors (2 points), methods - sample data (1 point), methods - missing data (1 point), methods - statistical analysis (5 points), methods - risk groups (1 point), methods - development and validation (1 point), results - participants (3 points), results - model development (2 points), results - model specification (2 points), results - model performance (1 point), results - model updating (1 point), discussion - limitations (1 point), discussion - interpretation (2 points), discussion - implications (1 point), supplementary information (1 point) and funding (1 point).

The individual TRIPOD checklists are contained in the Appendix section (Appendix 1-3) and the completed TRIPOD scoring for each article can be found in the Appendix section (Appendix 4-6).

Supplementary Table 1. Bias assessment of the studies included in the systematic review.

| **Author and year** | **TRIPOD score** | **TRIPOD ratio** |
| --- | --- | --- |
| Roy et al., 2020 | 18 | 0.49 |
| Gu et al., 2023 | 22 | 0.59 |
| Oyebode et al., 2020 | 11 | 0.30 |

Supplementary Table 2. TRIPOD Checklist.

| **Section/Topic** | **Item** |  | **Checklist Item** | **Page** |
| --- | --- | --- | --- | --- |
| **Title and abstract** | | | | |
| Title | 1 | D;V | Identify the study as developing and/or validating a multivariable prediction model, the target population, and the outcome to be predicted. | Section 1, Title page |
| Abstract | 2 | D;V | Provide a summary of objectives, study design, setting, participants, sample size, predictors, outcome, statistical analysis, results, and conclusions. | Section 2, Abstract |
| **Introduction** | | | | |
| Background and objectives | 3a | D;V | Explain the medical context (including whether diagnostic or prognostic) and rationale for developing or validating the multivariable prediction model, including references to existing models. | Section 3, Introduction |
|  | 3b | D;V | Specify the objectives, including whether the study describes the development or validation of the model or both. | Section 3, Introduction |
| **Methods** | | | | |
| Source of data | 4a | D;V | Describe the study design or source of data (e.g., randomized trial, cohort, or registry data), separately for the development and validation data sets, if applicable. | Section 4, paragraph 1 |
|  | 4b | D;V | Specify the key study dates, including start of accrual; end of accrual; and, if applicable, end of follow-up. | Section 4, paragraph 1 |
| Participants | 5a | D;V | Specify key elements of the study setting (e.g., primary care, secondary care, general population) including number and location of centres. | Section 4, paragraph 1 |
|  | 5b | D;V | Describe eligibility criteria for participants. | Section 4, paragraph 1 |
|  | 5c | D;V | Give details of treatments received, if relevant. | S_Text, paragraph 1 |
| Outcome | 6a | D;V | Clearly define the outcome that is predicted by the prediction model, including how and when assessed. | Section 4, paragraphs 2-4  S_Text, paragraphs 2-5 |
|  | 6b | D;V | Report any actions to blind assessment of the outcome to be predicted. | Not Applicable |
| Predictors | 7a | D;V | Clearly define all predictors used in developing or validating the multivariable prediction model, including how and when they were measured. | Section 4, paragraphs 2-4  S_Text, paragraphs 2-5 |
|  | 7b | D;V | Report any actions to blind assessment of predictors for the outcome and other predictors. | Not applicable |
| Sample size | 8 | D;V | Explain how the study size was arrived at. | Section 4, paragraphs 2-4  S_Text, paragraphs 2-5 |
| Missing data | 9 | D;V | Describe how missing data were handled (e.g., complete-case analysis, single imputation, multiple imputation) with details of any imputation method. | Section 4, paragraph 2 |
| Statistical analysis methods | 10a | D | Describe how predictors were handled in the analyses. | Section 4, paragraphs 2-4  S_Text, paragraphs 2-5 |
|  | 10b | D | Specify type of model, all model-building procedures (including any predictor selection), and method for internal validation. | Section 4, paragraphs 2-4  S_Text, paragraphs 2-5 |
|  | 10c | V | For validation, describe how the predictions were calculated. | Section 4, paragraphs 2-4  S_Text, paragraphs 2-5 |
|  | 10d | D;V | Specify all measures used to assess model performance and, if relevant, to compare multiple models. | Section 4, paragraphs 2-4  S_Text, paragraphs 2-5 |
|  | 10e | V | Describe any model updating (e.g., recalibration) arising from the validation, if done. | Section 4, paragraphs 2-4  S_Text, paragraphs 2-5 |
| Risk groups | 11 | D;V | Provide details on how risk groups were created, if done. | Section 4, paragraphs 2-4  S_Text, paragraphs 2-5 |
| Development vs. validation | 12 | V | For validation, identify any differences from the development data in setting, eligibility criteria, outcome, and predictors. | Section 4, paragraphs 2-4  S_Text, paragraphs 2-5 |
| **Results** | | | | |
| Participants | 13a | D;V | Describe the flow of participants through the study, including the number of participants with and without the outcome and, if applicable, a summary of the follow-up time. A diagram may be helpful. | Section 5, paragraph 1 |
|  | 13b | D;V | Describe the characteristics of the participants (basic demographics, clinical features, available predictors), including the number of participants with missing data for predictors and outcome. | Section 5, paragraph 1 |
|  | 13c | V | For validation, show a comparison with the development data of the distribution of important variables (demographics, predictors and outcome). | Section 5, paragraph 1 |
| Model development | 14a | D | Specify the number of participants and outcome events in each analysis. | Section 5, paragraphs 2-4 |
|  | 14b | D | If done, report the unadjusted association between each candidate predictor and outcome. | Section 5, paragraphs 2-4 |
| Model specification | 15a | D | Present the full prediction model to allow predictions for individuals (i.e., all regression coefficients, and model intercept or baseline survival at a given time point). | Section 5, paragraphs 2-4 |
|  | 15b | D | Explain how to the use the prediction model. | Section 5, paragraphs 2-4 |
| Model performance | 16 | D;V | Report performance measures (with CIs) for the prediction model. | Section 5, paragraphs 2-4 |
| Model-updating | 17 | V | If done, report the results from any model updating (i.e., model specification, model performance). | Section 5, paragraphs 2-4 |
| **Discussion** | | | | |
| Limitations | 18 | D;V | Discuss any limitations of the study (such as nonrepresentative sample, few events per predictor, missing data). | Section 6 |
| Interpretation | 19a | V | For validation, discuss the results with reference to performance in the development data, and any other validation data. | Section 6 |
|  | 19b | D;V | Give an overall interpretation of the results, considering objectives, limitations, results from similar studies, and other relevant evidence. | Section 6 |
| Implications | 20 | D;V | Discuss the potential clinical use of the model and implications for future research. | Section 6 |
| **Other information** | | | | |
| Supplementary information | 21 | D;V | Provide information about the availability of supplementary resources, such as study protocol, Web calculator, and data sets. | Online form |
| Funding | 22 | D;V | Give the source of funding and the role of the funders for the present study. | Online form |

Note: D;V – Development or Validation

Appendix 1 – Completed TRIPOD checklist for the ‘A machine learning approach predicts future risk to suicidal ideation from social media data’ article

| **Section/Topic** | **Item** |  | **Checklist Item** | **Page** |
| --- | --- | --- | --- | --- |
| **Title and abstract** | | | | |
| Title | 1 | D;V | Identify the study as developing and/or validating a multivariable prediction model, the target population, and the outcome to be predicted. | Page 1, 2 |
| Abstract | 2 | D;V | Provide a summary of objectives, study design, setting, participants, sample size, predictors, outcome, statistical analysis, results, and conclusions. | Page 1 |
| **Introduction** | | | | |
| Background and objectives | 3a | D;V | Explain the medical context (including whether diagnostic or prognostic) and rationale for developing or validating the multivariable prediction model, including references to existing models. | Page 1 |
|  | 3b | D;V | Specify the objectives, including whether the study describes the development or validation of the model or both. | Page 2 |
| **Methods** | | | | |
| Source of data | 4a | D;V | Describe the study design or source of data (e.g., randomized trial, cohort, or registry data), separately for the development and validation data sets, if applicable. | Page 9 |
|  | 4b | D;V | Specify the key study dates, including start of accrual; end of accrual; and, if applicable, end of follow-up. | Page 9 |
| Participants | 5a | D;V | Specify key elements of the study setting (e.g., primary care, secondary care, general population) including number and location of centres. | Page 9 |
|  | 5b | D;V | Describe eligibility criteria for participants. | Page 9 |
|  | 5c | D;V | Give details of treatments received, if relevant. | N/A |
| Outcome | 6a | D;V | Clearly define the outcome that is predicted by the prediction model, including how and when assessed. | Page 3, 4 |
|  | 6b | D;V | Report any actions to blind assessment of the outcome to be predicted. | N/A |
| Predictors | 7a | D;V | Clearly define all predictors used in developing or validating the multivariable prediction model, including how and when they were measured. | Page 3, 4 |
|  | 7b | D;V | Report any actions to blind assessment of predictors for the outcome and other predictors. | N/A |
| Sample size | 8 | D;V | Explain how the study size was arrived at. | Page 2, 9 |
| Missing data | 9 | D;V | Describe how missing data were handled (e.g., complete-case analysis, single imputation, multiple imputation) with details of any imputation method. | N/A |
| Statistical analysis methods | 10a | D | Describe how predictors were handled in the analyses. | Page 3 |
|  | 10b | D | Specify type of model, all model-building procedures (including any predictor selection), and method for internal validation. | Page 3 |
|  | 10c | V | For validation, describe how the predictions were calculated. | N/A |
|  | 10d | D;V | Specify all measures used to assess model performance and, if relevant, to compare multiple models. | N/A |
|  | 10e | V | Describe any model updating (e.g., recalibration) arising from the validation, if done. | N/A |
| Risk groups | 11 | D;V | Provide details on how risk groups were created, if done. | N/A |
| Development vs. validation | 12 | V | For validation, identify any differences from the development data in setting, eligibility criteria, outcome, and predictors. | N/A |
| **Results** | | | | |
| Participants | 13a | D;V | Describe the flow of participants through the study, including the number of participants with and without the outcome and, if applicable, a summary of the follow-up time. A diagram may be helpful. | Page 2, 3 |
|  | 13b | D;V | Describe the characteristics of the participants (basic demographics, clinical features, available predictors), including the number of participants with missing data for predictors and outcome. | N/A |
|  | 13c | V | For validation, show a comparison with the development data of the distribution of important variables (demographics, predictors and outcome). | N/A |
| Model development | 14a | D | Specify the number of participants and outcome events in each analysis. | Page 2, 3 |
|  | 14b | D | If done, report the unadjusted association between each candidate predictor and outcome. | N/A |
| Model specification | 15a | D | Present the full prediction model to allow predictions for individuals (i.e., all regression coefficients, and model intercept or baseline survival at a given time point). | N/A |
|  | 15b | D | Explain how to the use the prediction model. | N/A |
| Model performance | 16 | D;V | Report performance measures (with CIs) for the prediction model. | N/A |
| Model-updating | 17 | V | If done, report the results from any model updating (i.e., model specification, model performance). | N/A |
| **Discussion** | | | | |
| Limitations | 18 | D;V | Discuss any limitations of the study (such as nonrepresentative sample, few events per predictor, missing data). | Page 7, 8 |
| Interpretation | 19a | V | For validation, discuss the results with reference to performance in the development data, and any other validation data. | N/A |
|  | 19b | D;V | Give an overall interpretation of the results, considering objectives, limitations, results from similar studies, and other relevant evidence. | Page 7, 8 |
| Implications | 20 | D;V | Discuss the potential clinical use of the model and implications for future research. | Page 9 |
| **Other information** | | | | |
| Supplementary information | 21 | D;V | Provide information about the availability of supplementary resources, such as study protocol, Web calculator, and data sets. | N/A |
| Funding | 22 | D;V | Give the source of funding and the role of the funders for the present study. | N/A |

Appendix 2 - Completed TRIPOD checklist for the ‘An analysis of cognitive change in online mental health communities: A textual data analysis based on post replies of support seekers’ article

| **Section/Topic** | **Item** |  | **Checklist Item** | **Page** |
| --- | --- | --- | --- | --- |
| **Title and abstract** | | | | |
| Title | 1 | D;V | Identify the study as developing and/or validating a multivariable prediction model, the target population, and the outcome to be predicted. | Page 1 |
| Abstract | 2 | D;V | Provide a summary of objectives, study design, setting, participants, sample size, predictors, outcome, statistical analysis, results, and conclusions. | Page 3 |
| **Introduction** | | | | |
| Background and objectives | 3a | D;V | Explain the medical context (including whether diagnostic or prognostic) and rationale for developing or validating the multivariable prediction model, including references to existing models. | Page 4 |
|  | 3b | D;V | Specify the objectives, including whether the study describes the development or validation of the model or both. | Page 6, 7 |
| **Methods** | | | | |
| Source of data | 4a | D;V | Describe the study design or source of data (e.g., randomized trial, cohort, or registry data), separately for the development and validation data sets, if applicable. | Page 5 |
|  | 4b | D;V | Specify the key study dates, including start of accrual; end of accrual; and, if applicable, end of follow-up. | Page 5 |
| Participants | 5a | D;V | Specify key elements of the study setting (e.g., primary care, secondary care, general population) including number and location of centres. | Page 5 |
|  | 5b | D;V | Describe eligibility criteria for participants. | N/A |
|  | 5c | D;V | Give details of treatments received, if relevant. | N/A |
| Outcome | 6a | D;V | Clearly define the outcome that is predicted by the prediction model, including how and when assessed. | Page 10 |
|  | 6b | D;V | Report any actions to blind assessment of the outcome to be predicted. | N/A |
| Predictors | 7a | D;V | Clearly define all predictors used in developing or validating the multivariable prediction model, including how and when they were measured. | Page 11 |
|  | 7b | D;V | Report any actions to blind assessment of predictors for the outcome and other predictors. | N/A |
| Sample size | 8 | D;V | Explain how the study size was arrived at. | Page 5 |
| Missing data | 9 | D;V | Describe how missing data were handled (e.g., complete-case analysis, single imputation, multiple imputation) with details of any imputation method. | N/A |
| Statistical analysis methods | 10a | D | Describe how predictors were handled in the analyses. | Page 11 |
|  | 10b | D | Specify type of model, all model-building procedures (including any predictor selection), and method for internal validation. | Page 8 |
|  | 10c | V | For validation, describe how the predictions were calculated. | Page 8 |
|  | 10d | D;V | Specify all measures used to assess model performance and, if relevant, to compare multiple models. | Page 10 |
|  | 10e | V | Describe any model updating (e.g., recalibration) arising from the validation, if done. | Page 10 |
| Risk groups | 11 | D;V | Provide details on how risk groups were created, if done. | N/A |
| Development vs. validation | 12 | V | For validation, identify any differences from the development data in setting, eligibility criteria, outcome, and predictors. | N/A |
| **Results** | | | | |
| Participants | 13a | D;V | Describe the flow of participants through the study, including the number of participants with and without the outcome and, if applicable, a summary of the follow-up time. A diagram may be helpful. | Page 5 |
|  | 13b | D;V | Describe the characteristics of the participants (basic demographics, clinical features, available predictors), including the number of participants with missing data for predictors and outcome. | N/A |
|  | 13c | V | For validation, show a comparison with the development data of the distribution of important variables (demographics, predictors and outcome). | N/A |
| Model development | 14a | D | Specify the number of participants and outcome events in each analysis. | Page 5 |
|  | 14b | D | If done, report the unadjusted association between each candidate predictor and outcome. | N/A |
| Model specification | 15a | D | Present the full prediction model to allow predictions for individuals (i.e., all regression coefficients, and model intercept or baseline survival at a given time point). | Page 10, 11 |
|  | 15b | D | Explain how to the use the prediction model. | Page 8 |
| Model performance | 16 | D;V | Report performance measures (with CIs) for the prediction model. | N/A |
| Model-updating | 17 | V | If done, report the results from any model updating (i.e., model specification, model performance). | N/A |
| **Discussion** | | | | |
| Limitations | 18 | D;V | Discuss any limitations of the study (such as nonrepresentative sample, few events per predictor, missing data). | Page 12 |
| Interpretation | 19a | V | For validation, discuss the results with reference to performance in the development data, and any other validation data. | N/A |
|  | 19b | D;V | Give an overall interpretation of the results, considering objectives, limitations, results from similar studies, and other relevant evidence. | Page 11 |
| Implications | 20 | D;V | Discuss the potential clinical use of the model and implications for future research. | Page 11, 12 |
| **Other information** | | | | |
| Supplementary information | 21 | D;V | Provide information about the availability of supplementary resources, such as study protocol, Web calculator, and data sets. | N/A |
| Funding | 22 | D;V | Give the source of funding and the role of the funders for the present study. | N/A |

Appendix 3 - Completed TRIPOD checklist for the ‘Using machine learning and thematic analysis methods to evaluate mental health apps based on user reviews’ article

| **Section/Topic** | **Item** |  | **Checklist Item** | **Page** |
| --- | --- | --- | --- | --- |
| **Title and abstract** | | | | |
| Title | 1 | D;V | Identify the study as developing and/or validating a multivariable prediction model, the target population, and the outcome to be predicted. | Page 111141 |
| Abstract | 2 | D;V | Provide a summary of objectives, study design, setting, participants, sample size, predictors, outcome, statistical analysis, results, and conclusions. | Page 111142 |
| **Introduction** | | | | |
| Background and objectives | 3a | D;V | Explain the medical context (including whether diagnostic or prognostic) and rationale for developing or validating the multivariable prediction model, including references to existing models. | N/A |
|  | 3b | D;V | Specify the objectives, including whether the study describes the development or validation of the model or both. | Page 111143 |
| **Methods** | | | | |
| Source of data | 4a | D;V | Describe the study design or source of data (e.g., randomized trial, cohort, or registry data), separately for the development and validation data sets, if applicable. | Page 111142 |
|  | 4b | D;V | Specify the key study dates, including start of accrual; end of accrual; and, if applicable, end of follow-up. | N/A |
| Participants | 5a | D;V | Specify key elements of the study setting (e.g., primary care, secondary care, general population) including number and location of centres. | N/A |
|  | 5b | D;V | Describe eligibility criteria for participants. | N/A |
|  | 5c | D;V | Give details of treatments received, if relevant. | N/A |
| Outcome | 6a | D;V | Clearly define the outcome that is predicted by the prediction model, including how and when assessed. | Page 111144 |
|  | 6b | D;V | Report any actions to blind assessment of the outcome to be predicted. | N/A |
| Predictors | 7a | D;V | Clearly define all predictors used in developing or validating the multivariable prediction model, including how and when they were measured. | Page 111143 |
|  | 7b | D;V | Report any actions to blind assessment of predictors for the outcome and other predictors. | N/A |
| Sample size | 8 | D;V | Explain how the study size was arrived at. | Page 111143 |
| Missing data | 9 | D;V | Describe how missing data were handled (e.g., complete-case analysis, single imputation, multiple imputation) with details of any imputation method. | N/A |
| Statistical analysis methods | 10a | D | Describe how predictors were handled in the analyses. | N/A |
|  | 10b | D | Specify type of model, all model-building procedures (including any predictor selection), and method for internal validation. | Page 111143, 111144 |
|  | 10c | V | For validation, describe how the predictions were calculated. | Page 111144 |
|  | 10d | D;V | Specify all measures used to assess model performance and, if relevant, to compare multiple models. | Page 111144 |
|  | 10e | V | Describe any model updating (e.g., recalibration) arising from the validation, if done. | N/A |
| Risk groups | 11 | D;V | Provide details on how risk groups were created, if done. | N/A |
| Development vs. validation | 12 | V | For validation, identify any differences from the development data in setting, eligibility criteria, outcome, and predictors. | N/A |
| **Results** | | | | |
| Participants | 13a | D;V | Describe the flow of participants through the study, including the number of participants with and without the outcome and, if applicable, a summary of the follow-up time. A diagram may be helpful. | N/A |
|  | 13b | D;V | Describe the characteristics of the participants (basic demographics, clinical features, available predictors), including the number of participants with missing data for predictors and outcome. | N/A |
|  | 13c | V | For validation, show a comparison with the development data of the distribution of important variables (demographics, predictors and outcome). | N/A |
| Model development | 14a | D | Specify the number of participants and outcome events in each analysis. | N/A |
|  | 14b | D | If done, report the unadjusted association between each candidate predictor and outcome. | N/A |
| Model specification | 15a | D | Present the full prediction model to allow predictions for individuals (i.e., all regression coefficients, and model intercept or baseline survival at a given time point). | N/A |
|  | 15b | D | Explain how to the use the prediction model. | N/A |
| Model performance | 16 | D;V | Report performance measures (with CIs) for the prediction model. | N/A |
| Model-updating | 17 | V | If done, report the results from any model updating (i.e., model specification, model performance). | N/A |
| **Discussion** | | | | |
| Limitations | 18 | D;V | Discuss any limitations of the study (such as nonrepresentative sample, few events per predictor, missing data). | N/A |
| Interpretation | 19a | V | For validation, discuss the results with reference to performance in the development data, and any other validation data. | N/A |
|  | 19b | D;V | Give an overall interpretation of the results, considering objectives, limitations, results from similar studies, and other relevant evidence. | Page 111144 |
| Implications | 20 | D;V | Discuss the potential clinical use of the model and implications for future research. | N/A |
| **Other information** | | | | |
| Supplementary information | 21 | D;V | Provide information about the availability of supplementary resources, such as study protocol, Web calculator, and data sets. | N/A |
| Funding | 22 | D;V | Give the source of funding and the role of the funders for the present study. | N/A |

Appendix 4 - Completed TRIPOD scoring for the ‘A machine learning approach predicts future risk to suicidal ideation from social media data’ article

| Title - A machine learning approach predicts future risk to suicidal ideation from social media data |  |
| --- | --- |
| Author - Roy et al. |  |
| Feature | Score |
| title | 1 |
| abstract | 1 |
| introduction - background and objectives | 2 |
| methods - source of data | 2 |
| methods - participants | 2 |
| methods - outcome | 1 |
| methods - predictors | 1 |
| methods - sample data | 1 |
| methods - missing data | 0 |
| methods - statistical analysis | 2 |
| methods - risk groups | 0 |
| methods - development and validation | 0 |
| results - participants | 1 |
| results - model development | 1 |
| results - model specification | 0 |
| results - model performance | 0 |
| results - model updating | 0 |
| discussion - limitations | 1 |
| discussion - interpretation | 1 |
| discussion - implications | 1 |
| supplementary information | 0 |
| funding | 0 |
| Max score | 37 |
| Total score | 18 |
| TRIPOD ratio | 0.49 |

Appendix 5 - Completed TRIPOD scoring for the ‘An analysis of cognitive change in online mental health communities: A textual data analysis based on post replies of support seekers’ article

| Title - An analysis of cognitive change in online mental health communities: A textual data analysis based on post replies of support seekers |  |
| --- | --- |
| Author - Gu et al. |  |
| Feature | Score |
| title | 1 |
| abstract | 1 |
| introduction - background and objectives | 2 |
| methods - source of data | 2 |
| methods - participants | 1 |
| methods - outcome | 1 |
| methods - predictors | 1 |
| methods - sample data | 1 |
| methods - missing data | 0 |
| methods - statistical analysis | 5 |
| methods - risk groups | 0 |
| methods - development and validation | 0 |
| results - participants | 1 |
| results - model development | 1 |
| results - model specification | 2 |
| results - model performance | 0 |
| results - model updating | 0 |
| discussion - limitations | 1 |
| discussion - interpretation | 1 |
| discussion - implications | 1 |
| supplementary information | 0 |
| funding | 0 |
| Max score | 37 |
| Total score | 22 |
| TRIPOD ratio | 0.59 |

Appendix 6 - Completed TRIPOD scoring for the ‘Using machine learning and thematic analysis methods to evaluate mental health apps based on user reviews’ article

| Title - Using machine learning and thematic analysis methods to evaluate mental health apps based on user reviews |  |
| --- | --- |
| Author - Oyebode et al. |  |
| Feature | Score |
| title | 1 |
| abstract | 1 |
| introduction - background and objectives | 1 |
| methods - source of data | 1 |
| methods - participants | 0 |
| methods - outcome | 1 |
| methods - predictors | 1 |
| methods - sample data | 1 |
| methods - missing data | 0 |
| methods - statistical analysis | 3 |
| methods - risk groups | 0 |
| methods - development and validation | 0 |
| results - participants | 0 |
| results - model development | 0 |
| results - model specification | 0 |
| results - model performance | 0 |
| results - model updating | 0 |
| discussion - limitations | 0 |
| discussion - interpretation | 1 |
| discussion - implications | 0 |
| supplementary information | 0 |
| funding | 0 |
| Max score | 37 |
| Total score | 11 |
| TRIPOD ratio | 0.30 |

References

| [1] | G. Collins, J. Reitsma, D. Altman and K. Moons, “Transparent reporting of a multivariable prediction model for individual prognosis or diagnosis (TRIPOD): the TRIPOD statement,” Annals of internal medicine, vol. 162, no. 1, pp. 55-63, 2015. |
| --- | --- |
| [2] | H. Subramanian, R. Dey, W. Brim, N. Tillmanns, G. Cassinelli Petersen, A. Brackett, A. Mahajan, M. Johnson, A. Malhotra and M. Aboian, “Trends in development of novel machine learning methods for the identification of gliomas in datasets that include non-glioma images: a systematic review,” Frontiers in oncology, vol. 11, p. 788819, 2021. |
